# Cigarette smoke attenuates the production of cytokines by human plasmacytoid dendritic cells and enhances the release of IL-8 in response to TLR-9 stimulation

**DOI:** 10.1186/1465-9921-10-47

**Published:** 2009-06-10

**Authors:** Esmaeil Mortaz, Zsofia Lazar, Leo Koenderman, Aletta D Kraneveld, Frans P Nijkamp, Gert Folkerts

**Affiliations:** 1 Division of Pharmacology and Pathophysiology, Utrecht Institute for Pharmaceutical Sciences, Utrecht University, Utrecht, the Netherlands; 2 Department of Clinical Biochemistry, Faculty of Medical Sciences, Tarbiat Modares University, Tehran, Iran and Department of Basic Science, Section of Biochemistry, Faculty of Veterinary Medicine, Urmia University, Urmia, Iran; 3 Institute of Human Physiology and Clinical Experimental Research, Semmelweis University, 78/a Ulloi Str, Budapest, Hungary; 4 Department of Respiratory Medicine, University Medical Center Utrecht, Utrecht, the Netherlands

## Abstract

Myeloid and plasmacytoid dendritic cells (mDCs, pDC) are crucial to the immune system, detecting microorganisms and linking the innate and adaptive immunity. pDC are present in small quantities in tissues that are in contact with the external environment; mainly the skin, the inner lining of the nose, lungs, stomach and intestines. They produce large amounts of IFN-α after stimulation and are pivotal for the induction of antiviral responses. Chronic obstructive pulmonary disease (COPD) patients are known to be more susceptible to viral infections. We have demonstrated that exposure of mDC to cigarette smoke extract (CSE) leads to the release of chemokines, however, not much is known about the role of pDC in COPD. In this study, we addressed several key questions with respect to the mechanism of action of CSE on human pDC in an in vitro model. Human pDCs were isolated from normal healthy volunteers and subjected to fresh CSE and the levels of IL-8, TNF-α, IP-10, IL-6, IL-1, IL-12 and IL-10 and IFN-α were studied by both ELISA and real time PCR methods. We observed that CSE augmented the production of IL-8 and suppressed the release of TNF-α, IL-6 and IFN-α. Moreover, CSE suppressed PI3K/Akt signalling in pDC. In conclusion, our data indicate that CSE has both the potential to diminish anti-viral immunity by downregulating the release of IFN-α and other pro-inflammatory cytokines while, at the same time, augmenting the pathogenesis of COPD via an IL-8 induced recruitment of neutrophils.

## Introduction

Cigarette smoking is the most important risk factor for chronic obstructive pulmonary disease (COPD) and is expected to emerge as the third most common cause of death by 2020 [1,2]. Cigarette smoke induces both the release of chemokines from airway epithelial cells and alveolar macrophages resulting in the recruitment of neutrophils, monocytes, CD8^+ ^and CD4^+ ^cells into the lungs as well the release of excessive amounts of proteases from macrophages and neutrophils. Degradation of extracellular matrix components within alveolar walls by these proteases leads to the development of lung emphysema, the main characteristic of the COPD [1]. Despite a clear role for macrophages and neutrophils in the pathogenesis of emphysema, the contribution of dendritic cells (DCs) during COPD is not well documented.

In humans and mice, several subtypes of DC have been described. Generally, DCs can be divided into conventional DC (cDC or "myeloid" mDC) and plasmacytoid DC (pDC) [3-6]. While mDC are located mainly within tissues, human pDC represent a rare leukocyte subset that can be found in the peripheral blood and secondary lymphoid organs and are characterized by their plasma cell-like morphology and unique surface receptor phenotype [7]. Similar to mDC, pDCs sense pathogens via a repertoire of primary pattern recognition receptors (PRRs) including Toll-like receptors (TLRs). Unlike mDCs, however, pDCs express TLR9, which recognizes viral double-stranded microbial DNA [4]. This characteristic makes pDC a pivotal cell in innate antiviral immunity by allowing them to rapidly secrete abundant amounts of type I IFNs after exposure to various DNA and RNA viruses [7]

Current studies of the suppressive effects of cigarette smoke components on leukocyte function have shown some effects on mDCs. For example, it has been reported that exposure of human mDCs to CSE impairs the capacity of DCs to induce T-cell proliferation and Th1 differentiation while increasing Th2 differentiation, IL-10 production, and prostaglandin E2 release [8]. It is unknown which components of CSE are responsible for these effects, however, it is unlikely that these effects are mediated solely by nicotine because CSE is a mixture of thousands of compounds in which nicotine is present only in relatively low concentrations. In our study, we extended our CSE investigation to human pDC and examined the pDC chemokines, IL-8 and IP-10 as agents for attracting inflammatory cells [9]. The cytokines, TNF-α, IL-6, IL-1, IL-12 and IL-10, were also studied for their involvement in lymphocyte generation, local inflammatory responses, vasodilatation and damage of airway tissue [9].

## Materials and methods

### Reagents

CpG-A (ODN 2216) was purchased from InvivoGen (Toulouse, France), and CpG-C from HyCult Biotechnology (Uden, the Netherlands). RPMI 1640 and FCS were obtained from Gibco-BRL Life Technologies (Breda, Netherlands). LY294002, as PI3-K α inhibitor was purchased from Calbiochem (VWR International B.V. Amsterdam, The Netherlands).

### Preparation of CSE

Cigarette smoke extract (CSE) was prepared as described before[10]. CSE was generated by the burning of commercially available Lucky Strike cigarettes without filter (British-American Tobacco, Groningen, The Netherlands), using the TE-10z smoking machine (Teague Enterprises, Davis, CA), which is programmed to smoke cigarettes according to the Federal Trade Commission protocol (35-ml puff volume drawn for 2 s, once per minute [11]. Briefly, this machine was used to direct main and side stream smoke from one cigarette through 5 ml PBS. Hereafter, absorbance was measured spectrophotometrically and the buffer was standardized to a standard curve of CSE concentration against absorbance at 320 nm. The pH of the resultant extract was titrated to pH 7.4, and diluted with medium. This solution is considered to be 100% CSM. Solutions ranging from 0.75% to 1.5% were used in the present study following preliminary experiments, which indicated that these were nontoxic concentrations (viability ≥ 96%).

### Isolation of human plasmacytoid dendritic cells (pDCs)

Trisodium citrate (0.4% (w/v) (pH 7.4)) anti-coagulated fresh blood was obtained from healthy volunteers at the donor service of the University Medical Center (Utrecht, the Netherlands). Peripheral blood mononuclear cells (PBMC) were collected after centrifugation over isotonic Ficoll (Pharmacia, Uppsala, Sweden), and plasmacytoid dendritic cells were isolated from PBMCs by Diamond Plasmacytoid Dendritic Cell Isolation Kit by using CD304 MicroBeads (Magnetic cell sorting/MACS/; Miltenyi Biotech, Utrecht, the Netherlands). The purity was higher than 95% assessed by the flow cytometric analysis of CD123 expression (BD Biosciences, San Jose, CA, USA) in a pilot study. Cells were cultured in RPMI 1640 supplemented with 10% FCS.

### Activation of pDCs

Human pDCs were cultured with various stimuli at 37°C under a humidified atmosphere with 5% CO_2 _in a V-bottom 96-well plate for 5 h for real-time PCR analysis, 8 h for assessing cytokine production (10^6^/ml) and 90 min in the p-Akt assay (80,000 cells/well). Cells were stimulated with CSE (1.5%) alone and, in order to test the effect of CSE on activated pDCs, cells were simultaneously activated with TLR9 (2 μM CpG-A or 1 μM CpG-C) agonists. For the real time PCR analysis, cells were spun for 5 min at 1200 rpm at 4°C, washed once in cold PBS, the reverse transcription protocol was immediately, and cDNA was stored at -20°C till the real-time PCR reaction. For cytokine and chemokine measurements, supernatants were collected by spinning for 5 min at 1200 rpm at 4°C and stored at -20°C till analysis.

### Assessing cell viability

Cell viability was assessed on fresh cells at the end of each treatment with propidium idodide (PI, Sigma-Alderich) staining. 10,000 events were counted in the cell gate by FACS (FACS Calibur, BD Bioscience, Becton Dickinson).

### Measurement of cytokines

IFN-α was measured by sandwich ELISA (PBL Biomedical Laboratories, Piscataway, NY, USA) while IL-6, IL-8, TNF-α, IL-10 and IL-1β concentrations were measured with the Cytometric Bead Array Human Inflammation Kit (BD Bioscience) on a flow cytometer (FACS Calibur).

### Real-time PCR

cDNA synthesis and Q-PCR were performed using the Superscript III Platinum CellsDirect Two-step qRT-PCR Kit with SYBR Green (Invitrogen, Toulouse, France) according to the manufacturer's instructions. Briefly, after incubation cells were washed once and resuspended in 10 μl cold PBS and 2 μl (i.e. 10,000 cells) was lysed in 11 μl lysis buffer. Then the cell lysate was treated with DNase I to degrade any contaminating DNA. During first strand cDNA synthesis, the reaction mix contained 2 μl RT Enzyme Mix (100 units/μl SuperScript III RT, 20 units/μl RNaseOUT Recombinant Ribonuclease Inhibitor), 20 μl 2× RT Reaction mix (2.5 μM oligo(dT)_20_, 2.5 ng/μl random hexamer, 10 mM MgCl_2 _and dNTPs) in 40 μl reaction volume. The reaction was allowed to proceed (incubation: 25°C, 10 min, RT: 50°C, 20 min; PCT-100 Programmable Thermal Controller, MJ Research Inc., Watertown, MA, USA), and then it was halted by heating the samples to 85°C for 5 min. Afterwards 1 μl of RNase H (2 U/μl) was added to each well and incubated at 37°C for 20 min. The reaction was chilled on ice.

Primers for Q-PCR were synthesized by Invitrogen and were as follows: β_2_-microglobulin (forward, 5'-CTCCGTGGCCTTAGCTGTG-3'; reverse, 5'-TTTGGAGTACGCTGGATAGCCT-3'), IL-8 (forward 5'-CTGGCCGTGGCTCTCTTG-3'; reverse 5'-CCTTGGCAAAACTGCACCTT-3'), IFN-α (forward 5'-GGTGCTCAGCTGCAAGTCAA-3'; reverse, 5'-GCTACCCAGGCTGTGGGTT-3'). The Q-PCR reactions were performed in 25 μl reaction volume with 4 μl cDNA, 12.5 μl Platinum SYBR Green qPCR SuperMix-UDG and 200 nM sense and antisense primers. Cycling conditions were 50°C for 2 minutes, 95°C for 2 minutes and then 50 cycles of 95°C for 15 seconds and 60°C for 30 seconds (ABI PRISM 7000 Sequence Detection System, Applied Biosystems, Foster City, CA, USA). PCR amplicon efficiency for primers was calculated using serially diluted pooled cDNA. The relative level of mRNA expression of a specific gene was calculated based on ΔΔCT method, and normalized to mRNA level for the housekeeping gene β_2_-microglobulin.

### Measurement of Nitric Oxide

As an indicator of NO production, nitrite concentration was measured in the supernatants of samples. Equal volumes of the sample and Griess reagent (1% sulphanilamide in 5% phosphoric acid and 0.1% *N*-[1-naphthyl]-ethylendiamine) were mixed and the absorbance was read at 450 nm as described before [12]. The amount of nitrite was obtained by an extrapolation from a standard curve with sodium nitrite and was expressed as μmol/mg tissue.

### Detection of p-Akt by flow cytometry

The method was adopted from Guiducci et al [13]. 8 × 10^4 ^pDCs were pretreated with LY294002 (10 μM) and then stimulated with 1 μM CpG-C, CSE or the combination of the two stimulants for 90 min in a V-bottom 96-well plate. Then cells were fixed with 4% paraformaldehyde for 15 min at 37°C. Cells were then washed with incubation buffer (1% FCS in PBS), and permeabilized with 90% ice-cold methanol for 30 min on ice. After washing cells were blocked for 10 min in incubation buffer, and then stained with Alexa Fluor 647 anti-human AKT (pS473) or with Alexa 647 isotype control antibodies according to the manufacturer's instructions. All antibodies were purchased from Cell Signaling Technologies (Bioke, Leiden, the Netherlands).

### Statistical analysis

For assessing the effect of CSE on IFN-α production induced by different stimulants, unpaired t-test was used. In other comparisons, one-way ANOVA was applied with the Newman-Keuls Multiple Comparison post-hoc test (GraphPad Prism 4.0; GraphPad Software Inc., San Diego, California, USA). Data are expressed as mean ± SEM, p < 0.05 was considered to be significant.

## Results

### CSE induced IL-8 release is enhanced by TLR9 stimulation

**Figure 1:**
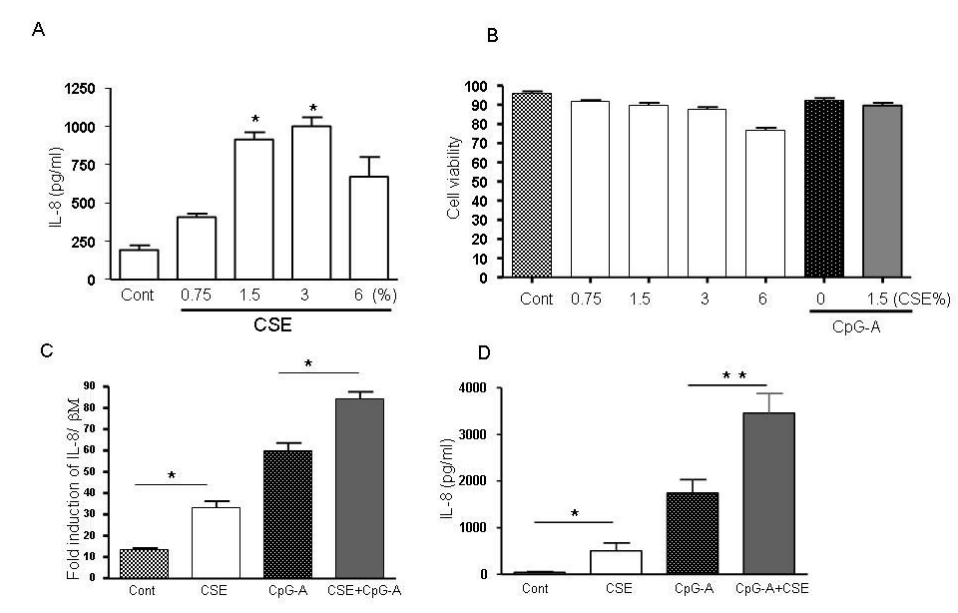
**CSE induces IL-8 release and potentiates TLR-mediated IL-8 release**. pDCs (10^6 ^cells/ml) were stimulated with various concentrations of CSE (0.75–6%) for 8 h and amounts of IL-8 release was determined by supernatants of samples by ELISA (A). The viability of cells were determined after treatment with CSE at various concentration, CpG-A (2 μM) or in combination CSE (1.5%) and CpG-A (2 μM) by staining of cells with propidium idodide (PI) for 5 min. Cells were then analyzed by flow cytometry for a cell count of 10000. Data are presented as means ± SD from triplicates and three independent experiments (B). pDC (10^6 ^cells/ml) were stimulated with CSE (1.5%), CpG-A (2 μM) or in combination for 5 and 8 h and amounts of IL-8 at mRNA levels (C) and protein levels (D) was determined by total RNA and supernatants of samples by real time PCR and ELISA, respectively. Data presented mean ± SEM of 3 independent experiments; *p < 0.05, **p < 0.01).

The effect of CSE on IL-8 production by pDCs was studied. Cells were exposed for 5 h and 8 h to different concentrations of CSE (0.75–6%). The level of IL-8 was determined by Q-PCR and ELISA, respectively. Incubation of pDC with CSE dose-dependently induced release of IL-8 (Fig. 1A) with an optimum at 1.5% CSE. At high concentration (6%), the release of IL-8 was declined which is likely due to the cytotoxicity of CSE at high concentrations as detected by PI staining (Fig. 1B). As shown in Fig. 1, stimulation of cells with CpG-A (2 μM) induces up-regulation of the mRNA and protein release of IL-8 and addition of CSE (1.5%) with CpG-A potentiates the mRNA expression and protein releases of IL-8 (Fig. 1C and 1D). No significant cytotoxicity of cells were seen either with CpG-A alone or in combination with CSE (1.5%) as detected by PI staining (Fig. 1B).

### TLR9 induced cytokines release is inhibited by CSE

**Figure 2:**
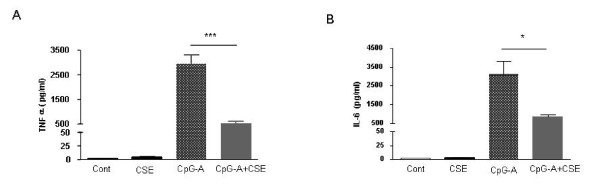
**CSE suppresses TNF-α and IL-6 production induced by CpG-A of pDC**. pDCs (10^6 ^cells/ml) were stimulated with CSE (1.5%), CpG-A (2 μM) or the in combination for 8 h for protein levels (A and B). Cytokines concentration of cell supernatants were measured with cytometric bead array (mean ± SEM of 3 independent experiments; *p < 0.05, ***p < 0.001).

We studied the effects of CSE on cytokine release of human pDC. TLR9 stimulation by CpG-A resulted in the production of TNF-α and IL-6 (Fig. 2A and 2B). In contrast, CSE exposure of CpG-A-activated pDC resulted in a profound reduction in TNF-α and IL-6 production when compared to pDC exposed only to CpG-A (Fig. 2A and 2B). CSE did not have an effect on IL-10, IL-1β, IP-10 and IL-12 release by pDC (data not shown).

### CSE inhibited TLR-9-induced IFN-α at mRNA and protein levels of pDC

**Figure 3:**
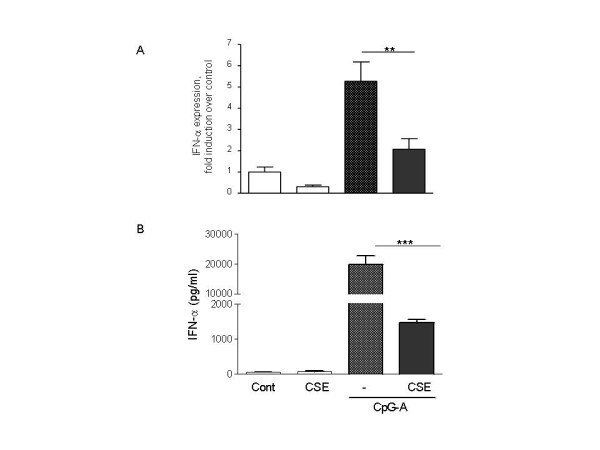
**CSE inhibits TLR-induced mRNA expression and protein production of IFN-α by pDC**. pDCs (10^6^cells/ml) were stimulated with CpG-A (2 μM), CSE (1.5%) or in combination for 5 h, then lyzed, followed by RNA extraction and real time PCR analysis. IFN-α mRNA expression is normalized to housekeeping gene (β_2_-microglobulin) expression, and shown as fold induction over expression of non-stimulated control cells (A). pDCs (1 × 10^6^/ml) were stimulated with CpG-A (2 μM), CSE (1.5%) or incombination and CSE (1.5%) for 8 h (B). IFN-α concentration of culture supernatants were determined by ELISA (mean ± SEM of 3 independent experiments; ***p < 0.001).

To test whether CSE modulates IFN-α mRNA expression and protein release, pDCs were exposed to CSE alone or in combination with TLR9 agonist for 5 h and 8 h for determination mRNA and protein levels, respectively. CpG-A has been shown to rapidly induced high levels of IFN-α from human pDCs [14]. CSE did not up-regulate mRNA expression and protein release of IFN-α and suppressed the mRNA and protein levels of IFN-α in CpG-A activated cells (Fig. 3A and 3B).

### CSE suppressed TLR9-induced IFN-α induction in pDC via attenuating the PI3K/Akt pathway

**Figure 4:**
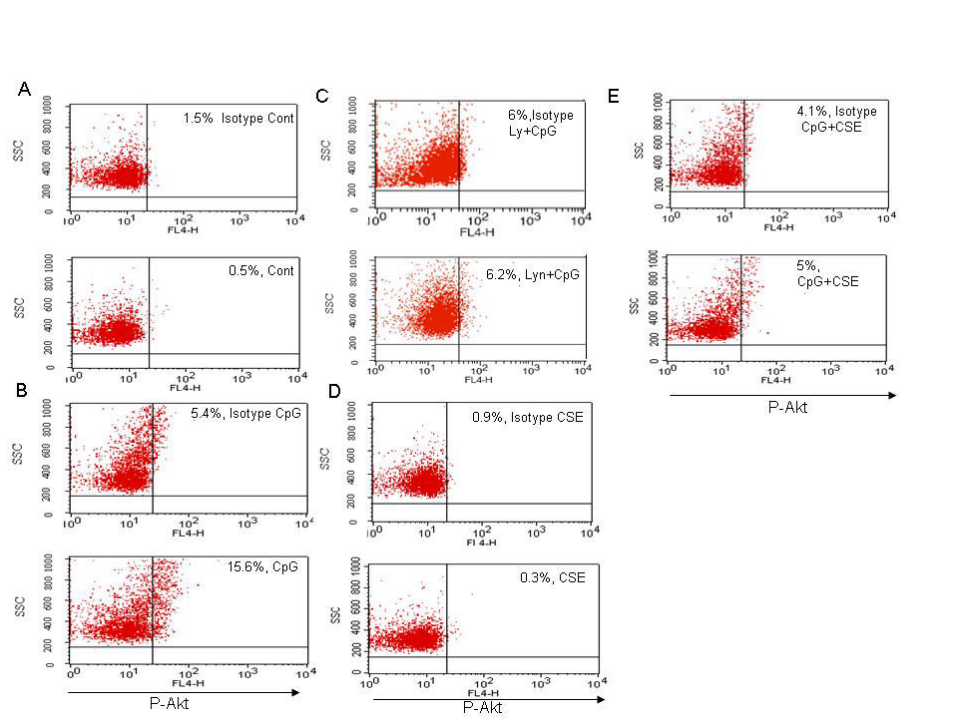
**CSE inhibits Akt activation induced by CpG-C by human pDCs**. pDCs (10^6^cells/ml) were cultured for 90 min (A) without stimulant as control, (B) stimulated with CpG-C (1 μM), (C) pretreatment for 30 with LY294002 (10 μM) and then stimulated with CpG-C (1 μM), (D) stimulated with CSE (1.5%) and (E) in combination CSE (1.5%) and CpG-C (1 μM). Following fixation and permeabalization, cells were stained with Alexa Fluor 647 phospho anti-human Akt or Alexa 647 isotype control antibodies, as specified in the "Materials and methods". A representative of three independent experiments is shown. 3,000 events were counted in the pDC gate.

Recently, it has been demonstrated that CpG ODN induced production of type I IFN-α by pDC, induced is highly dependent on the activity of phosphatidylinositol-3 kinase (PI3K) [13]. We measured phosphorylated Akt (p-Akt), a downstream molecules of PI3K signaling [15]. CpG-C as a positive control, induced phosphorylation of Akt after 90 min compared to control cells (Fig. 4A and 4B), Ly 294002 treatment (as a specific inhibitor for PI3K pathway) suppressed the phosphorylation of Akt (Fig 4C). CSE did not induce phosphorylation of Akt (Fig. 4D) by itself, but suppressed the phosphorylation of Akt induced by CpG-C (Fig. 4E).

## Discussion

COPD is a major cause of morbidity and mortality worldwide. There is increasing evidence that implicates viral infections as a major risk factor for exacerbations of COPD [16,17]. Dendritic cells are the main sentinels of the immune systems. They are capable of detecting virus-associated nucleic acids through PRRs such as TLRs and cytosolic viral sensors. Viral nucleic acids activate DCs to produce a variety of soluble factors including proinflammatory cytokines and type I interferons (IFNs). pDCs are particularly skilled in virus detection and have been shown to produce high levels of IFN-α/β and TNF-α in response to a wide variety of viruses in both humans and mice [3]. Given that cigarette smoking is associated with an increased risk of infections, we hypothesized that cigarette smoke may modulate the expression and release of pDC-associated mediators. Therefore, we analyzed the effect of CSE on pDC with an in vitro experimental model. CSE induced the release of IL-8 and suppressed the TLR9-ligand induced TNF-α, IL-6 and IFN-α production. Moreover, the suppressive effects of CSE were associated with an attenuated phosphorylation of Akt. To our knowledge, these data are the first that demonstrate modulatory effects of CSE on pDC function.

Cigarette smoke contains an extraordinarily complex mixture of chemicals [18]. As a result, identifying cigarette smoke constituents responsible for certain biological effects is a daunting task, leading some to favour a reductionist approach over the use of complete extracts from cigarette smoke. Although CSE is arguably not fully representative of "true" cigarette smoke exposure, it is important to note that in vivo cells are not exposed to cigarette smoke, but rather to cigarette smoke constituents that have been solubilised into biological fluids such as the epithelial and alveolar lining fluid in the lungs. In recognition of these caveats, we used a number of parallel and complimentary approaches to define the effect of soluble cigarette smoke on different inflammatory cells [19-21].

Accumulating evidence supports that IL-8 is the main chemokine in the recruitment of neutrophils into the airways and lung of COPD patients [22,23]. We and others have demonstrated that CSE can induce the release of IL-8 (or CXCL-8) from human monocytes, mDC and murine mast cells [10,20]. In the current study, CSE induced IL-8 release by pDC and, interestingly, this effect was more pronounced after adding a TLR9 agonist. This finding has also been reported in human mDC exposed to a combination of CSE and LPS (a TLR4 ligand) [8]. Thus, we can postulate that CXCL-8 production by pDC in response to CSE may have a significant impact on the recruitment of pro-inflammatory cells in to the airway. As stated earlier, CSE did not induce the production of TNF-α, IL-6 and IL-10, IL-1β (data not shown) and IFN-α production, but suppressed the the ability of a TLR9 agonist (CpG-A and CpG-C) to induce these cytokines. Suppressive effects of CSE on cytokine production have been shown in other cell types [24-27]. For example, CSE inhibits the release of TNF-α but not IL-6 after stimulation with Poly I:C as TLR3 ligand in PBMC [28].

The inhibition of IFN-α production by CSE has been earlier demonstrated in experiments with human lung fibroblast and epithelial cells [29]. Although the inhibition of IFN-α production could be a general effect of CSE, it can also be speculated that CSE is having a critical effect on pDC as this cell type produces high amounts of IFN-α. Thus CSE may play an important role in the suppression of the antiviral cytokine production of smokers and COPD patients. To further understand the effects of CSE on the attenuation of IFN-α secretion, molecular biology approaches were used. To date, only a few studies have documented ways in which INF-α by pDC is regulated. For example, IL-10 suppresses IFN-α secretion independently of TLR9 by pDC [30] and cross-linking surface complexes of IgE/FcεRI or pre-treatment of pDC with TNF-α reduces INF-α release in response to CpG-A via an autocrine loop of TNF-α [30] In the current study, we did not find any release of TNF-α and IL-10 by CSE (data not shown). Other studies have shown that CSE decreases the expression of IFN-α regulatory factor-7 (IRF-7) transcripts [29] and that exogenous nitric oxide (NO) inhibits IFN-α production of pDC by downregulating IRF-7 expression via a cGMP-dependent pathway [31]. In our study, we can exclude the effects of NO since we did not find the production of TNF-α and NO by CSE (data not shown).

Earlier studies have demonstrated that PI3K (Akt) plays a critical role in production of IFN-α by human pDC and that the downregulation of this pathway with pharmacologic inhibitors abrogates the release of IFN-α [13,32]. Similarly, we have shown that CSE is also capable of attenuating Akt (down-stream molecules of PI3k) phosphorylation leading to reductions in IFN-α production. Besides controlling IFN-α expression, the PI3K pathway is also involved in a variety of biological processes, including cell survival and proliferation, B and T cell receptor signaling, and the activation of G protein-coupled receptors, such as chemokine receptors [33]. The PI3K pathway is also activated by various TLR ligands and can function as a positive or negative regulator of TLR responses depending on the cell type and the TLR ligand used [34]. PI3K activation is an important early step in the signaling pathway leading to IRF-7 nuclear translocation and type I IFN-α production after TLR 9 activation by pDCs that differentially regulate the IRF-7 and NF-κB signaling pathways [13].

pDCs, through their production of high levels of type I IFN-α, play a critical role in the pathophysiology of various autoimmune diseases, such as systemic lupus erythematosus, psoriasis, or Sjögren's disease [35-37]. However, our studies are purely in vitro and should be verified by in vivo investigations before translating their relevance to these diseases.

In conclusion, CSE induces both the release of IL-8 and the suppression of TLR9 ligand-induced IFN-α and pro-inflammatory cytokine production. Increased release of IL-8 may lead to a magnified recruitment of neutrophils into the lungs. On the other hand, suppression of IFN-α may lead to impaired host defence thereby increasing the risk of airway infections, a major cause of COPD exacerbation. As downregulation of the PI3K pathway is likely responsible for these effects, stimulators of the PI3K pathway may have a protective effect in COPD patients and lead to the development of new drugs to treat COPD.

## Abbreviations

APC: Antigen Presenting Cells; 7AAD: 7-Amino-Actinomycin D; BDCA: Blood Dendritic Cell Antigen; CSE: Cigarette Smoke Extract; DC: Dendritic Cells; ELISA: Enzyme-Linked Immunosorbant Assay; FACS: Fluorescence Activated Cell Scanner; FITC: Fluorescein Isothiocyanate; PMA: Phorbol 12-Myristate 13-Acetate; PI: Propidium Iodide; PI3k: Phosphatidylinositol-3 kinase; PBMC: Peripheral Blood Mononuclear Cells.

## Competing interests

The authors declare that they have no competing interests.

## Authors' contributions

EM conceived the study, and participated in the design of the study and performed immunoassays, FACS analysis, statistical analysis, and wrote the first draft and final version of the manuscript. ZL carried out the ELISAs and biochemical experiments. LK and AK took part in critical revision of the manuscript. FPN participated in the design and coordination of the study. GF conceived of study, and participated in the design of the study and supervised the project. All authors read and approved the final manuscript.
